# Dismantling the curse of the “first”

**DOI:** 10.1016/j.isci.2023.106054

**Published:** 2023-02-16

**Authors:** Aja M. Nicely

**Affiliations:** 1 The University of Texas at Austin College of Natural Sciences, Austin, TX, USA

## Main text

**Figure:**
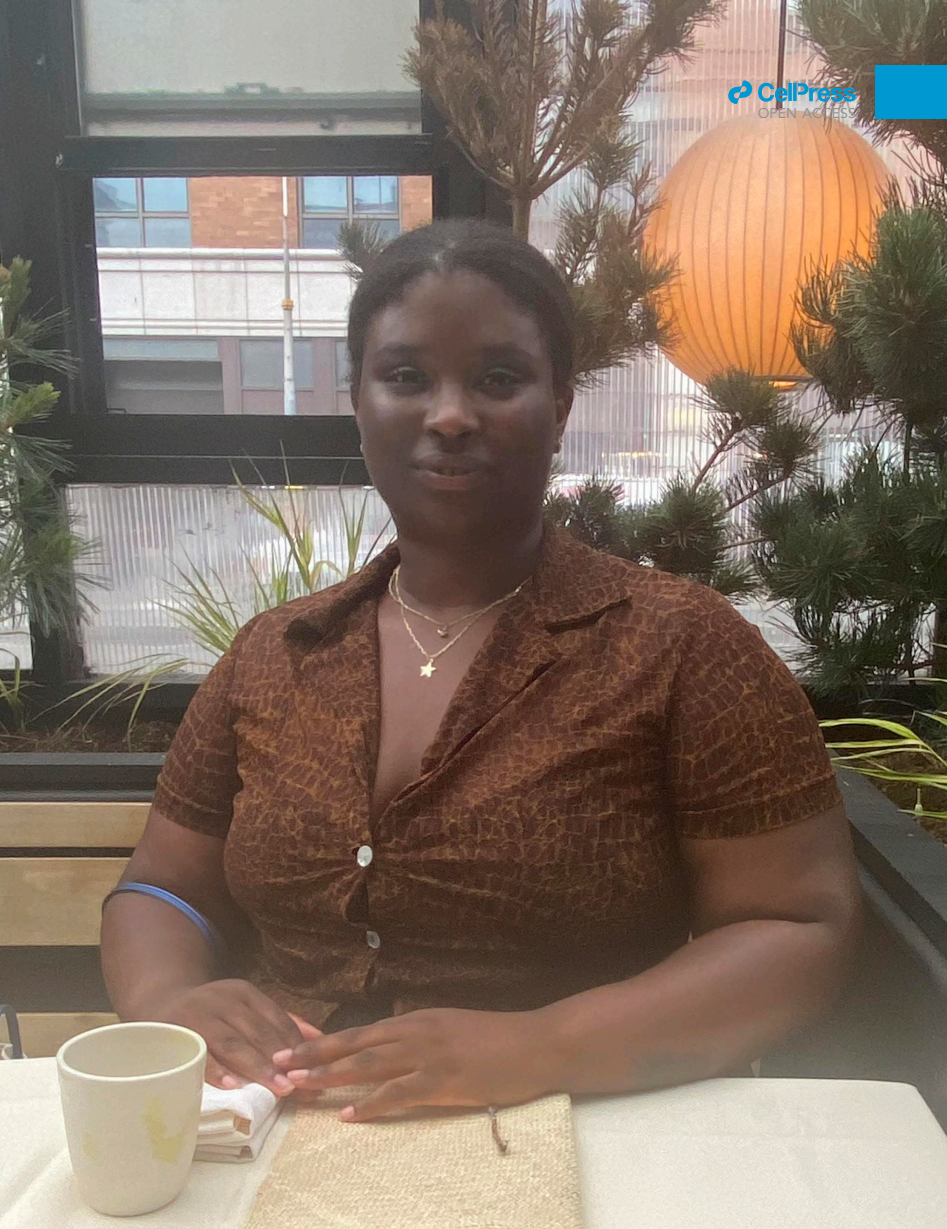

For a discipline often lauded for its ubiquity, science can be incredibly isolating. This is especially true for Black folks. When I was in third grade, my favorite teacher Mrs. Schiele, a Black woman herself, tasked our class with individual reports concerning a Black historical figure not often acknowledged in the mainstream. For me, she chose, Dr. Mae C. Jemison, the first Black woman to travel to space. When I got home, I immediately accused my parents of hiding valuable information from me, “How could you not tell me about this?! Black people in space!” I obsessively read articles and interviews about and of Dr. Jemison; learning of a Black woman in science changed my life. I wanted to become someone as brilliant as her. My teacher and my parents affirmed me in this desire. “You *are* brilliant. You *can* do it,” they would say to me. I never thought that I could not be a scientist until I actually began pursuing science. I realized how few people looked like me, and even fewer believed in me. In science, not belonging is often disguised by the descriptors “first”, “only”, and “one of”. Knowing one’s own ability and brilliance is difficult when you are faced with the overwhelming solitude of science. Having a community of Black scientists will alleviate that burden for many young researchers.

I find joy in establishing a community of chemists from underrepresented backgrounds. Many of my efforts have been toward increasing the accessibility of undergraduate research opportunities. During my own undergraduate experience, I felt extremely isolated and had little help in securing a research position. In a large school like University of Texas, research positions are an even more finite resource. Without research experience, students will not pursue higher positions within academia or scientific careers. This lack of representation does a disservice to Black students and the field of chemical research. By expanding the inclusion of the TEJAS program at University of Texas at Austin (UT), I have helped even the playing field. This program provides research opportunities for undergraduate students who receive work-study accommodations. This has been incredibly beneficial because students of lower socioeconomic backgrounds are prioritized, and those students are historically underrepresented in science. When I was able to obtain a summer undergraduate research position, my work was unpaid. I had two other jobs to afford living, minimizing my time in lab. Instead, students in the TEJAS program, one of which is working in my own research lab, can focus on enjoying their research and learning. To provide additional resources for undergraduates, I have acted as a counselor for the Jackson School of Geosciences Virtual Math and Science Institute, which provides courses for minority high school students before they began their collegiate careers. This offers students with resources to be successful in “weed-out” STEM courses. These programs also allow these students to develop community with one another. Upon completion of these programs, students have a community of professors, graduate students, and other undergraduates to look to when they need encouragement or assistance.

I also find joy in my work. Initially, I coped with my own lack of community by dreams of how I could contribute to improving the world with my research. I was inspired to pursue chemistry after learning about the pathology and treatment of various diseases in both my Cancer Biology and Technology course and my Drug Design, Development, and Delivery course. Learning how the work of researchers directly saves lives led to my pursuit of an organic chemistry and methodology PhD at UT. Currently, I work with developing rapid assembly of drug molecule analogs, particularly making carbon–nitrogen bonds. Amine-containing molecules make up about 75% of market drug products, yet they are extremely challenging to synthesize. To make healthcare accessible, there must be an efficient and inexpensive route to life-saving drug molecules. Many diseases often disproportionately affect Black and brown communities. While researchers diligently work to discover cures, serving these communities and ensuring Black and Brown people are properly treated is usually not a priority. I plan to work on improving the equity and transparency of the pharmaceutical industry. With my work as a medicinal chemist, I will leverage my skills and discoveries to lobby for access to healthcare for underserved communities.

While I have been lucky to have the unconditional support of my family and friends, forging my path in science is still something I have largely done alone. This has made me a self-sufficient and diligent chemist, who has a passion for establishing equity and diversity in every space I occupy. I still would have preferred to have more support within my scientific network. That is my hope for future Black scientists. Science is important and challenging work; we should not have to do it alone.

